# Excess Body Mass Index and Risk of Liver Cancer: A Nonlinear Dose-Response Meta-Analysis of Prospective Studies

**DOI:** 10.1371/journal.pone.0044522

**Published:** 2012-09-18

**Authors:** Rui Rui, Jiao Lou, Li Zou, Rong Zhong, Ji Wang, Ding Xia, Qi Wang, Heng Li, Jing Wu, Xuzai Lu, Chuanqi Li, Li Liu, Jiahong Xia, Hua Xu

**Affiliations:** 1 Department of Urology, Tongji Hospital, Tongji Medical College, Huazhong University of Science and Technology, Wuhan, China; 2 Department of Epidemiology and Biostatistics and MOE Key Lab of Environment and Health, School of Public Health, Tongji Medical College, Huazhong University of Science and Technology, Wuhan, China; 3 Department of Urology and Helen-Diller Comprehensive Cancer Center, University of California San Francisco, San Francisco, California, United States of America; 4 School of Medicine and Health Management, Tongji Medical College, Huazhong University of Science and Technology, Wuhan, China; 5 Department of Epidemiology and Biostatistics and Guangdong Key Lab of Molecular Epidemiology, Guangdong Pharmaceutical University, Guangzhou, China; 6 Department of Cardiovascular Surgery, Union Hospital, Huazhong University of Science and Technology, Wuhan, China; MOE Key Laboratory of Environment and Health, School of Public Health, Tongji Medical College, Huazhong University of Science and Technology, China

## Abstract

**Background:**

Excess body weight measured as body mass index (BMI) has a positive association with risk of common cancers. However, previous meta-analyses related to BMI and liver cancer had inconsistent results. The purpose of the current study is to establish a nonlinear dose-response relationship between BMI and incidence risk of liver cancer.

**Methods:**

A systematic literature search for relevant articles published from 1966 to November 2011 was conducted in PUBMED and EMBASE digital databases. Additional articles were manually searched by using the reference lists of identified papers. Restricted cubic splines and generalized least-squares regression methods were used to model a potential curvilinear relationship and to make a dose-response meta-analysis. Stratified analysis, sensitivity analysis and assessment of bias were performed in our meta-analysis.

**Results:**

8 articles including 1,779,471 cohort individuals were brought into meta-analysis. A non-linear dose-response association between BMI and risk of liver cancer was visually significant (*P* for nonlinearity<0.001), besides, the point value of BMI also enhanced the results quantitatively, where relative risks were 1.02 (95%CI = 1.02–1.03), 1.35 (95%CI = 1.24–1.47) and 2.22-fold (95%CI = 1.74–2.83) when BMI was at the point of 25, 30 and 35 kg/m^2^ compared with reference (the median value of the lowest category), respectively. The ethnicity of the population was found as the main source of heterogeneity. In subsequent stratified analysis, no evidence of heterogeneity was showed in Asian and White populations (*P* for heterogeneity>0.1), and all value of BMI still presented significantly increased risk of cancer.

**Conclusions:**

The findings from meta-analysis provided that excess BMI had significant increased association with risk of liver cancer, although the biological mechanisms underlying the obesity-cancer link still need to be clarified.

## Introduction

Primary liver cancer is the sixth most common cancer in the world, with an estimated incidence of 564,000 per year [1]. Since most patients in early of liver cancer have no obvious symptoms, liver cancer is usually diagnosed in the later stages and has a dismal prognosis with an average 5-year survival rate of 6–11%, making it the third leading cause of cancer mortality worldwide [2]. Given that there are no established screening methods for early detection and no better treatments to prolong survival time, the identification of modifiable risk factors for the primary prevention against liver cancer is of considerable public health importance.

Epidemiological studies have identified chronic Hepatitis B or C viral (HBV or HCV) infection, alcohol consumption, cigarette smoking, and aflatoxin exposure as important risk factors for liver cancer [2]–[4]. Meanwhile, other suspected risk factors, such as obesity, diet, insulin resistance, use of oral contraceptives, and iron overload, still need to be clarified [5], of which, obesity [Body mass index, (BMI) ≥30 kg/m^2^] has been raised by accumulating evidence to be associated with the increased risk of liver cancer [6], [7]. In the context of cirrhosis, obesity has been an independent risk factor for the development of hepatocellular carcinoma [4]; moreover, diabetes, as a closely correlated factor with obesity in the composition of metabolic syndrome, has been suggested to be independent risk factor of liver cancer [8]. All these evidences suggested a key role of obesity associated with liver cancer.

However, reports on the association between obesity and risk of liver cancer are still controversial. For instance, a large-scale in Asia-Pacific [9] showed no clear associations between BMI and risk of liver cancer, as well as other three studies [10]–[12] in Australia, Japan and the Unite State, respectively. Even the three meta-analyses so far performed on obesity and liver cancer risk also yielded inconsistent results. In the two meta-analyses on both liver cancer incidence and mortality suggested that BMI was associated with an increased risk of liver cancer in both males and females [13], [14]; whereas the third meta-analysis of liver cancer incidence, Renehan et al. found no significant association [15]. The divergent results between these meta-analyses may due to the nonlinear association between BMI with liver cancer risk and the heterogeneity by merging liver cancer mortality and incidence together. Although it has been hypothesized that excess BMI can increase liver cancer risk, none of the previous meta-analyses have examined the shape of the dose–response relationship by conducting nonlinear dose response analyses. Considering recent evidence showed a nonlinear relation between BMI and pancreatic cancer, we hypothesized a nonlinear association also existed in BMI and liver cancer risk [16]. As a result, we summarized the published prospective studies on incidence risk of liver cancer to update the previous meta-analysis and performed a dose-response meta-analysis as described by Larsson et al. [17] to investigate a potential nonlinear association between BMI and liver cancer risk.

## Materials and Methods

### Search strategy and selection criteria

A systematic literature search for relevant articles on body mass index (BMI) in humans and risk of liver cancer published from 1966 to November 2011 was conducted in PUBMED and EMBASE databases with a search strategy that combined text words: “obesity, weight, overweight, body mass index, BMI or body size” and “liver cancer, liver tumor or hepatocellular carcinoma” without language restriction. In addition, we manually searched the reference lists of retrieved articles to identify additional articles.

Studies were eligible for inclusion in this meta-analysis if they satisfied the following criteria: 1) the prospective study evaluated the relationship between BMI and the risk of liver cancer; 2) the outcome was liver cancer incidence; 3) the exposure of interest was the BMI for 3 or more quantitative categorized level; 4) the study reported relative risks with 95% confidence intervals (CIs) for each BMI category. Studies on gallbladder cancer, hepatic adenomas, and other subtypes of liver cancer were excluded. When multiple studies shared the same population, only the one with the most detailed information of outcome or the largest sample size was included.

### Data extraction

Data were extracted independently and in duplicate by 2 reviewers (RUI and LOU). For each study, the following data were extracted from the published article: first author's name, publication year, study location, ethnicity of population, follow-up years, age, measure method of BMI, sample size of gender, BMI categories and risk estimate for each BMI category, and covariates adjusted for multivariable analysis. Unless otherwise indicated, ethnicity was assumed to correspond to the geographic location where subjects were selected. We extracted the relative risks with their 95% CIs that reflected the greatest degree of adjustment for potential confounders.

For every study, the midpoint of the upper and lower boundaries in each category was assigned as median BMI to each corresponding relative risk. If the upper boundary for the highest category was not reported (such as BMI ≥30 kg/m^2^), the range of the highest boundary was set as the same amplitude as the adjacent category [17]. The categories of BMI<18.5 were excluded as a convenient way to evaluate the association between excess BMI and liver cancer, and then, the midpoint of the lowest category was regarded as a reference level.

### Statistical analysis

A dose-response meta-analysis was performed to examine a potential nonlinear relationship between BMI and liver cancer. We used restricted cubic splines with 3 knots at fixed 5%, 50% and 95% to model a potential curvilinear relationship [18] and then a dose-response meta-analysis was conducted using generalized least-squares regression, taking into account the trend estimation of summarized data across multiple studies as described by Orsini et al [19]. The method requires the distribution of case and person-years and median level of BMI in each category to the corresponding relative risk for each study (the relative risks with estimates for at least 3 quantitative exposure categories are known). Between-study heterogeneity was assessed by the *Q* statistic test. Heterogeneity was considered significant when *P*<0.1 for the *Q* statistic [20]. DerSimonian and Laird random-effects models [21] were applied when heterogeneity was significant; otherwise, a fixed-effects model was applied. The pooled relative risks for specific exposure values were finally estimated (per 0.5 kg/m^2^ increase from BMI = 18.5 to 35 kg/m^2^) using a procedure described by Orsini and Greenland compared with the lowest exposure value of BMI [22]. A test for a nonlinearity relationship between BMI and liver cancer was calculated by making the second spline equal to zero [17]. To test and verify the non-linear model, in separate analyses, we pooled the relative risks for comparable categories of BMI as compared with the lowest category under common linear method, classifying the BMI into 3 groups: 18.5–25 kg/m^2^ (reference), 25–30 kg/m^2^, and ≥30 kg/m^2^. To explore potential sources of between-study heterogeneity, further stratified analyses, if feasible, were conducted by gender (male, female or combined male and female), ethnicity of population (Asian or White), measured methods for BMI (measured or self-reported BMI), separately. Additionally, sensitivity analysis was performed by omitting each article in turn to determine the influence of each study on the overall estimate [23]. Publication bias was evaluated by Egger's test [24]. All statistical analyses were performed in STATA software (version 10.1), and a 2-sided *P* value of less than 0.05 was considered significant.

## Results

### Studies characteristics

As shown by Figure S1, 35 of 1,372 initial articles were identified for full-text review. The reports by Jee et al. and Oh et al. shared the overlapping samples, and the report by Jee et al. was selected with completed information [25], [26]. Five articles of mortality [12], [27]–[30] and another four articles [31]–[34] included in previous meta-analyses were excluded for insufficient data of BMI category. Finally, a total of 8 articles with 12 studies met the inclusion criteria to be included in our meta-analysis [10], [11], [26], [35]–[39].

All articles were published in English and more than half of the articles (6/8) were published after the year 2006. Analyses of relative risks for BMI were performed separately for males and females in 2 articles [10], [26] and thus were considered as independent studies. The general population, HCV-based and HBV-based populations in the article by Chen et al. were also considered separately [35]. In total, this meta-analysis included 12 independent prospective studies of 1, 779, 471 cohort individuals, the durations of follow-up varied from 3.6 years to 19 years across the included studies (Table 1). 8 studies were performed for Asian, and 4 studies were for White population. 10 studies collected measured BMI, and 2 study collected self-reported data. Most studies provided relative risk estimates that were adjusted by age (n = 12), cigarette smoking (n = 10), and alcohol consumption (n = 5). Additionally, 3 cohort studies were conducted in persons with cirrhosis [36], [37], [39].

**Table 1:** Characteristics for prospective studies of body mass index and risk of liver cancer included in a meta-analysis.

Study	P,	Location	Race^b^	F,	Age	MM^d^	No. of case		BMI	Relative risk	Adjustments
	year^a^			year^c^			Male	Female	(kg/m^2^)	with 95% CI	
Kuriyama	2005	Japan	A	9	>40	S	69	31		Male:	Age, smoking,
[10]									18.5–24.9	1.00 Reference	meat, vegetables,
									25.0–27.4	1.30 (0.54, 3.16)	alcohol intake,
									27.5–29.9	0.91 (0.30, 2.80)	bean-paste soup,
										Female:	type of health
									18.5–24.9	1.00 Reference	insurance
									25.0–27.4	0.80 (0.40, 1.63)	
									27.5–29.9	1.14 (0.46, 2.87)	
Rapp [11]	2005	Austria	W	9.9	Male:	M	57			Male:	Age, smoking,
					41.8				18.5–24.9	1.00 Reference	occupational
					Female				25.0–29.9	1.32 (0.73, 2.37)	group
					42.5				30–34.9	1.67 (0.75, 3.72)	
Jee [26]	2008	Korean	A	10.8	Male:	M	8759	1761		Male:	Age, gender,
					45.0				<20	0.90 (0.81, 1.00)	smoking status
					Female				20–22.9	0.97 (0.90, 1.04)	
					49.4				23.0–24.9	1.00 Reference	
									25.0–29.9	1.04 (0.96, 1.13)	
									≥30	1.63 (1.27, 2.10)	
										Female:	
									<20	0.85 (0.67, 1.06)	
									20–22.9	0.76 (0.64, 0.91)	
									23.0–24.9	1.00 Reference	
									25.0–29.9	1.14 (0.97, 1.35)	
									≥30	1.39 (1.00, 1.94)	
Chen [35]	2008	Taiwan	A	14	NA	M	222	69		HBV(−), HCV(−):	Age, education,
									<23	1.00 Reference	gender, smoking,
									23–24.9	0.88 (0.41, 1.86)	alcohol intake
									25–29.9	0.86 (0.42, 1.74)	
									≥30	2.36 (0.91, 6.17)	
										HBV(+), HCV(−):	
									<23	1.00 Reference	
									23–24.9	1.40 (0.97, 2.02)	
									25–29.9	1.17 (0.81, 1.69)	
									≥30	1.36 (0.64, 2.89)	
										HBV(−), HCV(+):	
									<23	1.00 Reference	
									23–24.9	1.05 (0.41, 2.73)	
									25–29.9	3.02 (1.48, 6.14)	
									≥30	4.13 (1.38, 12.4)	
Ioannou^e^	2007	USA	W	3.6	NA	M	Total: 100		Total:	Age, HCV	
[36]									<25	1.00 Reference	infection, HBsAg
									25–30	2.80 (1.40, 5.40)	
									≥30	2.50 (1.30, 4.90)	
Nkontchou^e^	2006	France	W	4.2	61.4	M	Total: 220		Total:	Age,sex, diabetes,	
[37]									<25	1.00 Reference	cirrhosis cause,
									25–30	2.00 (1.40, 2.70)	alcohol intake,
									≥30	2.80 (2.00, 4.00)	hepatitis infection
											status
Samanic	2006	Sweden	W	19	34.3	M	194			Male:	Age, smoking,
[38]									18.5–24.9	1.00 Reference	calendar year
									25.0–29.9	1.45 (1.06, 1.98)	
									>30	3.13 (2.04, 4.79)	
Yu^e^	2008	Taiwan	A	14.7	18–60	M	134			Male:	Age, cigarette
[39]									<18.5	1.55 (0.49, 4.93)	smoking, alcohol
									18.5–24.9	1.00 Reference	consumption,
									25.0–29.9	1.48 (1.04, 2.12)	history of diabetes
									≥30	1.96 (0.72, 1.64)	

Abbreviations: NA, no available; HBV (+)/(−), hepatitis b virus infection positive/negative; HCV (+)/(−), hepatitis c virus infection positive/negative.

a P, years means Publication year.

b A refers to Asian and W refers to White.

c F, years means Follow-up years.

d MM means Measure Methods, M refer to measured BMI directly and S refers to self-reported BMI.

e Cohort studies were conducted in patients with cirrhosis cohort.

### Overall dose-response association between BMI and risk of liver cancer

**Figure 1:**
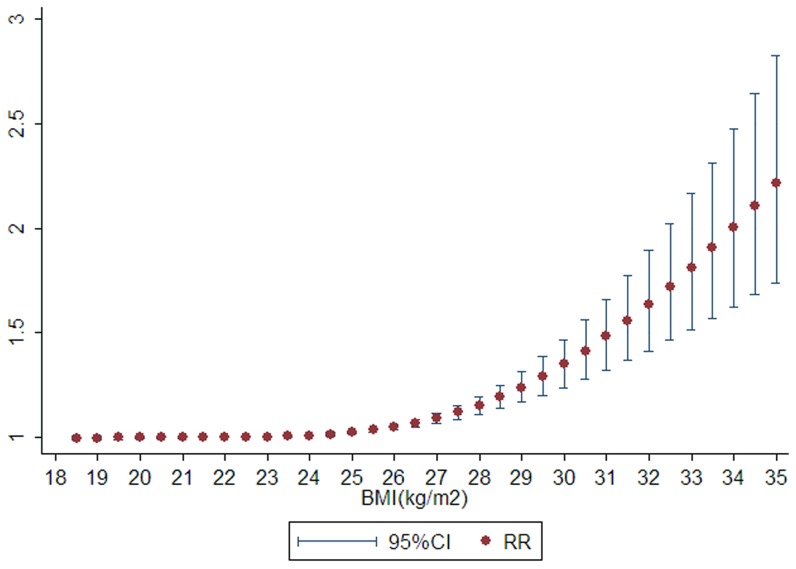
Nonlinear dose-response analyses of body mass index and relative risk of liver cancer in meta-analysis. (*P*-heterogeneity = 0.001; *P*-non-linear<0.001).

**Figure 2:**
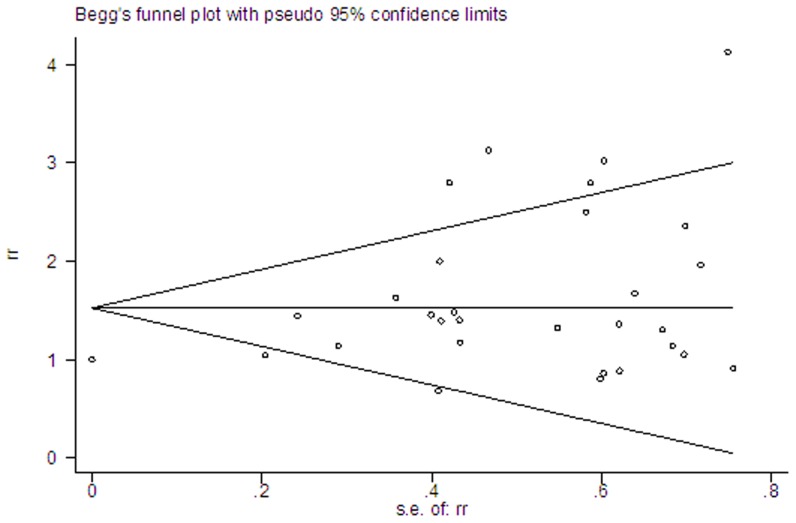
Publication bias in the studies included in this meta-analysis.

Significant heterogeneity was observed across studies (*P* for heterogeneity <0.001), and thus the random-effects model was applied. There was a significant nonlinear dose-response association visually between BMI and risk of liver cancer (*P* for nonlinear<0.001, Figure 1) with a significantly increased trend of relative risk as per 0.5 kg/m^2^ increase in BMI. Several representative point value enhanced the association: the point estimate of BMI at 25 kg/m^2^ had a modest increased risk of liver cancer 1.02 (95% CI = 1.02–1.03), while 30 kg/m^2^ and 35 kg/m^2^ both conferred significantly increased cancer relative risks of 1.35 (95%CI = 1.24–1.47) and 2.22 (95%CI = 1.74–2.83), indicating a significant and evolutionary risk of liver cancer along with BMI increasing. Egger's test showed a modest publication bias, with a *P* value of 0.03 (Figure 2).

When we pooled the relative risks for comparable categories of BMI in a separate analysis under linear model, the relative risks of liver cancer were 1.13 (95%CI = 1.05–1.21) for BMI = 25–30 kg/m^2^ and 2.09 (95%CI = 1.72–2.45) for BMI ≥30 kg/m^2^, suggesting the linear method present the similar trend as our non-linear results.

After excluding the 3 articles of cirrhosis cohort, a significant nonlinear dose-response relationship was still found between BMI and risk of liver cancer (*P* for nonlinear<0.001), the relative risks were 1.01 (95%CI = 1.00–1.02), 1.27 (95%CI = 1.17–1.38) and 2.10 (95%CI = 1.61–2.74) at 25, 30 and 35 kg/m^2^, respectively.

### Subgroup analyses between BMI and the risk of liver cancer

Specific data for the association between BMI and liver cancer were stratified on the basis of sex into 2 subgroups: studies on males (n = 6) and females (n = 2). In males, between-study heterogeneity still existed (*P* for heterogeneity <0.001). A significant dose-response effect was observed for BMI with liver cancer risk in male (*P* for nonlinear <0.001; Figure S2), and the point value of BMI at 30 and 35 kg/m^2^ yielded significant relative risks of 1.51 (95%CI = 1.35–1.70) and 2.57 (95%CI = 1.95–3.38). However, there were insufficient female samples to form restricted cubic splines to establish nonlinear model, so the stratified analysis on females failed.

**Figure 3:**
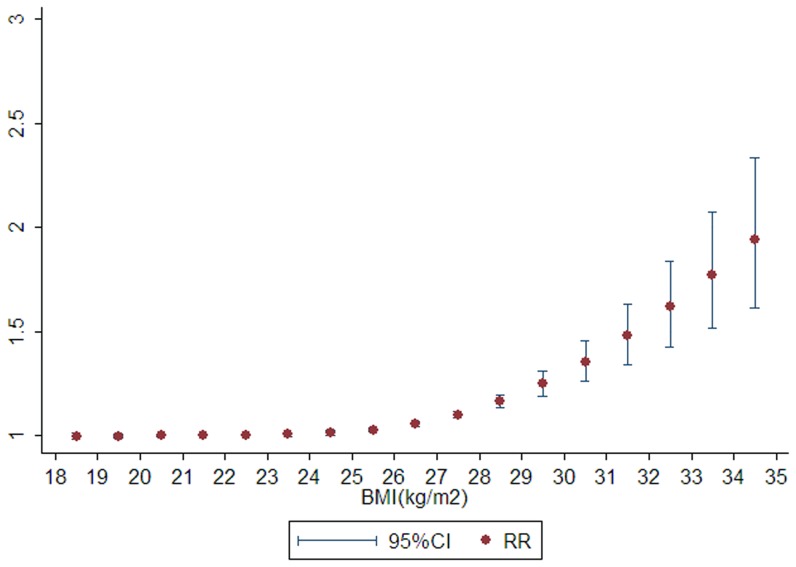
The curve of body mass index and relative risk of liver cancer in Asian population.

**Figure 4:**
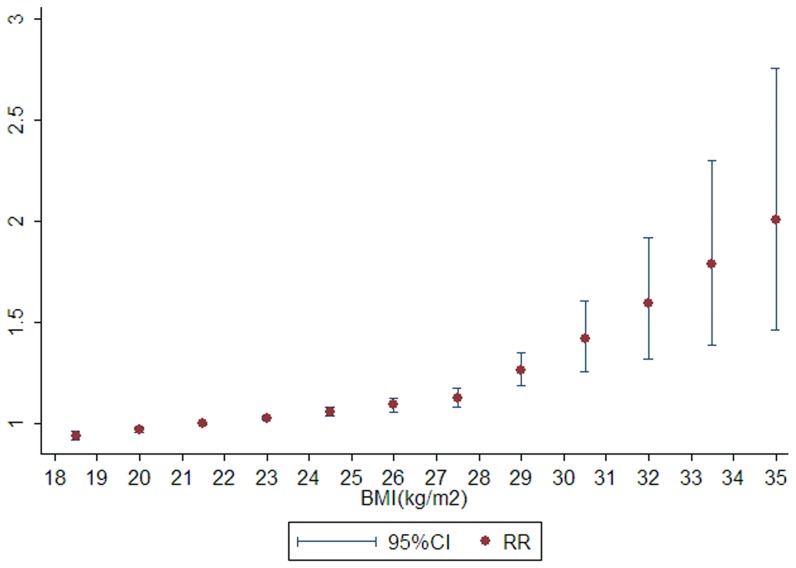
The curve of body mass index and relative risk of liver cancer in White population.

After stratifying by ethnicity (Figure 3 and Figure 4), heterogeneity was totally removed by Asian and White subgroup, and a significant nonlinear dose-response relationship between BMI and risk of liver cancer was observed in both ethnicity (*P* for nonlinear <0.001; *P* for heterogeneity = 0.470 and 0.119, receptively).

According to the methods of obtaining BMI, analysis was only performed in the subgroup of reporting measured BMI. Despite the nonlinear relationship existed (*P* for nonlinear <0.001), there no statistically significance between BMI and liver cancer under measured BMI subgroup with significant heterogeneity (*P* for heterogeneity = 0.032) (Figure S3).

### Sensitivity analysis

Considering the presentation of heterogeneity, sensitivity analysis was performed by excluding each report in turn. Before and after removal of each report, a significant nonlinear dose-response relationship between BMI and risk of liver cancer still remained and the point estimation of BMI at 25, 30, and 35 kg/m^2^ did not substantially change (Table 2), suggesting a high stability for the current result.

**Table 2:** Relative risk with 95% CI of body mass index (kg/m^2^) at point of 25, 30 and 35 in comparison with reference (median value of lowest category) by omitting each article in sensitivity analysis.

Omitted study	BMI = 25 kg/m^2^		BMI = 30 kg/m^2^		BMI = 35 kg/m^2^		*P* *	*P* **
	RR	95%CI	RR	95%CI	RR	95%CI		
Kuriyama [10]	1.02	1.02–1.03	1.33	1.23–1.44	2.15	1.72–2.70	<0.001	<0.001
Rapp [11]	1.02	1.02–1.03	1.34	1.24–1.45	2.18	1.73–2.74	<0.001	<0.001
Jee [26]	1.01	1.01–1.02	1.29	1.20–1.38	2.11	1.69–2.63	<0.001	<0.001
Chen [35]	1.02	1.01–1.03	1.34	1.24–1.45	2.18	1.73–2.74	<0.001	<0.001
Ioannou [36]	1.02	1.02–1.03	1.36	1.25–1.47	2.27	1.79–2.87	<0.001	<0.001
Nkontchou [37]	1.02	1.01–1.03	1.31	1.21–1.43	2.07	1.62–2.65	<0.001	<0.001
Samanic [38]	1.02	1.01–1.03	1.30	1.20–1.41	2.00	1.57–2.53	<0.001	<0.001
Yu [39]	1.04	1.02–1.05	1.30	1.17–1.44	1.90	1.38–2.62	<0.001	<0.001

Abbreviation: BMI, body mass index; RR, relative risk; CI, confidence interval.

* *P* values for heterogeneity.

** *P* values for nonlinear.

## Discussion

Previously, to summarize the publications on excess BMI and risk of liver cancer, two meta-analyses have been conducted, both based on the data assessing liver cancer incidence and mortality, and another meta-analysis focused on liver cancer incidence included five articles. In this current work, 12 prospective studies on the incidence of liver cancer were included. Then a dose-response meta-analysis was applied by increasing every 0.5 kg/m^2^ from the value of BMI = 18.5 to fit an accurate relationship with risk of liver cancer. Therefore, current findings produced a persuasive comprehending conclusion that a non-linear dose-response association existed between BMI and incidence risk of liver cancer: the greater increased by the value of BMI, the higher risk produced by liver cancer. Several representative point value enhanced the association: not only was the point of BMI at 35 kg/m^2^ associated with liver cancer, with the highest 2.22-fold risk (95%CI = 1.74–2.83), but also BMI equal to 30 kg/m2 still yielded a 1.35-fold (95%CI = 1.24–1.47) increased risk compared with reference BMI (the median value of lowest category).

Significant heterogeneity existed in our meta-analysis as expected. We examined in more detail which factors contributed to significant heterogeneity by sensitivity and stratified analysis. The ethnic background appeared to be the critical sources of heterogeneity. Obesity has increased in many North American and European populations over the past three decades; more than 21.9% adults in the United States and the United Kingdom were obese, and the rates for Asian population generally lagged behind [40]. In our meta-analysis, White population had higher risk than Asian at same point of BMI. The possible explanation might be that the White had a higher percentage of body fat per BMI category than individuals of Asian. Even in the same ethnic group, BMI-body-fat association showed heterogeneity [41]. This was an important consideration given that the correlation between BMI and risk of liver cancer might differ across diverse ethnic backgrounds.

The previous meta-analyses all indicated that males were at higher risk of developing liver cancer than females, however, due to insufficient female samples to form restricted cubic splines to establish nonlinear model, we only found the significant association between BMI with liver cancer in males. The results of males came to a similar estimate as SC Larsson: relative risks were 2.42 (95%CI = 1.83–3.20) and 2.46 (95%CI = 1.91–3.18), respectively. The possible reason reflected the gender difference might relate to the differences of hormone. Epidemiologic and animal studies have suggested that men have a higher incidence of liver cancer than women might be due to the stimulatory effects of androgen and the protective effects of estrogen [42]. A meta-analysis found that testosterone concentration were associated with a lower risk of diabetes in male but a higher risk in female [43]. Diabetes, as a closely correlated factor with obesity in metabolic syndrome, has been suggested to be independent risk factor of liver cancer. So the effect of hormone might be the reason for the stronger association of obesity with liver cancer in male than in female. The real causes for the gender difference still need to be explored.

There were also several limitations that might affect the interpretation of the results in our meta-analysis. First of all, restricted cubic splines required sufficient data to form polynomial models within each category of BMI. Unfortunately, there were insufficient data to permit a dose-response relationship in female, different ethnic group and measure method subgroups, affecting the power of the statistics. Secondly, some important confounders have not been measured with sufficient precision. Only some article had considered alcohol consumption, cigarette smoking, hepatitis infection status, dietary factors and physical activity. Lack of adjustment for these important risk factors limited the ability to generalize between obesity and liver cancer. Finally, BMI was the most commonly reported but not the best index to assess body adiposity; other anthropometric measures methods such as waist-hip ratio and waist circumference [44] have been suggested to be more pertinent and sensitive disease predictors than BMI [45] However, few studies provided such information to permit comprehensive analysis of associations across studies.

Considerable evidence has accumulated to support links between obesity and increased risk of several types of cancer: postmenopausal breast, endometrium, kidney, colon, pancreas, gall-bladder and esophageal cancers [46], but the mechanisms linking both obesity and these common cancers are not yet fully understood. Recent studies have suggested that inflammation may be the primary potential mechanism linking obesity with liver cancer. Biological evidence indicated that high levels of interleukin 6 and tumor necrosis factor, which are associated with obesity, turned healthy cells into malignant ones through chronic low-grade inflammation of the liver [47]. Moreover, inflammation was just one possible link between obesity and cancer. In addition, alcohol consumption, hepatitis viruses and many other factors probably also affected these cells [48]. Diabetes as an independent risk factor of liver cancer [8] was closely correlated with obesity. Both diabetes and obesity contribute to metabolic syndrome [49]. Some hypotheses have indicated that obesity is involved in metabolic abnormalities [50], in which insulin and insulin-like growth factors may distort the normal balance between determinants of cell proliferation, differentiation, and apoptosis, and thus may promote carcinogenesis [51]. Therefore, the biological mechanisms underlying the obesity-cancer link still need to be clarified.

### Conclusions

The findings from meta-analysis provided accurate depiction of the role of BMI with liver cancer, suggesting that excess body mass index were associated with risk of liver cancer. Further, the correlation might differ across diverse ethnic backgrounds.

## Supporting Information

Figure S1 **Flow chart of study selection.**
(TIF)

Figure S2 **The curve of body mass index and risk of liver cancer for male (** ***P*** **-heterogeneity = 0.005;** ***P*** **-non-linear<0.001).**
(TIF)

Figure S3 **The curve of body mass index and risk of liver cancer for directly measure BMI (** ***P*** **-heterogeneity = 0.032;** ***P*** **-non-linear<0.001).**
(TIF)
