# Supplementary figures and images for: Excess Body Mass Index and Risk of Liver Cancer: A Nonlinear Dose-Response Meta-Analysis of Prospective Studies
Source: PLoS One. 2012 Sep 18;7(9):e44522. doi: 10.1371/journal.pone.0044522 (PMC3445525; doi:10.1371/journal.pone.0044522)

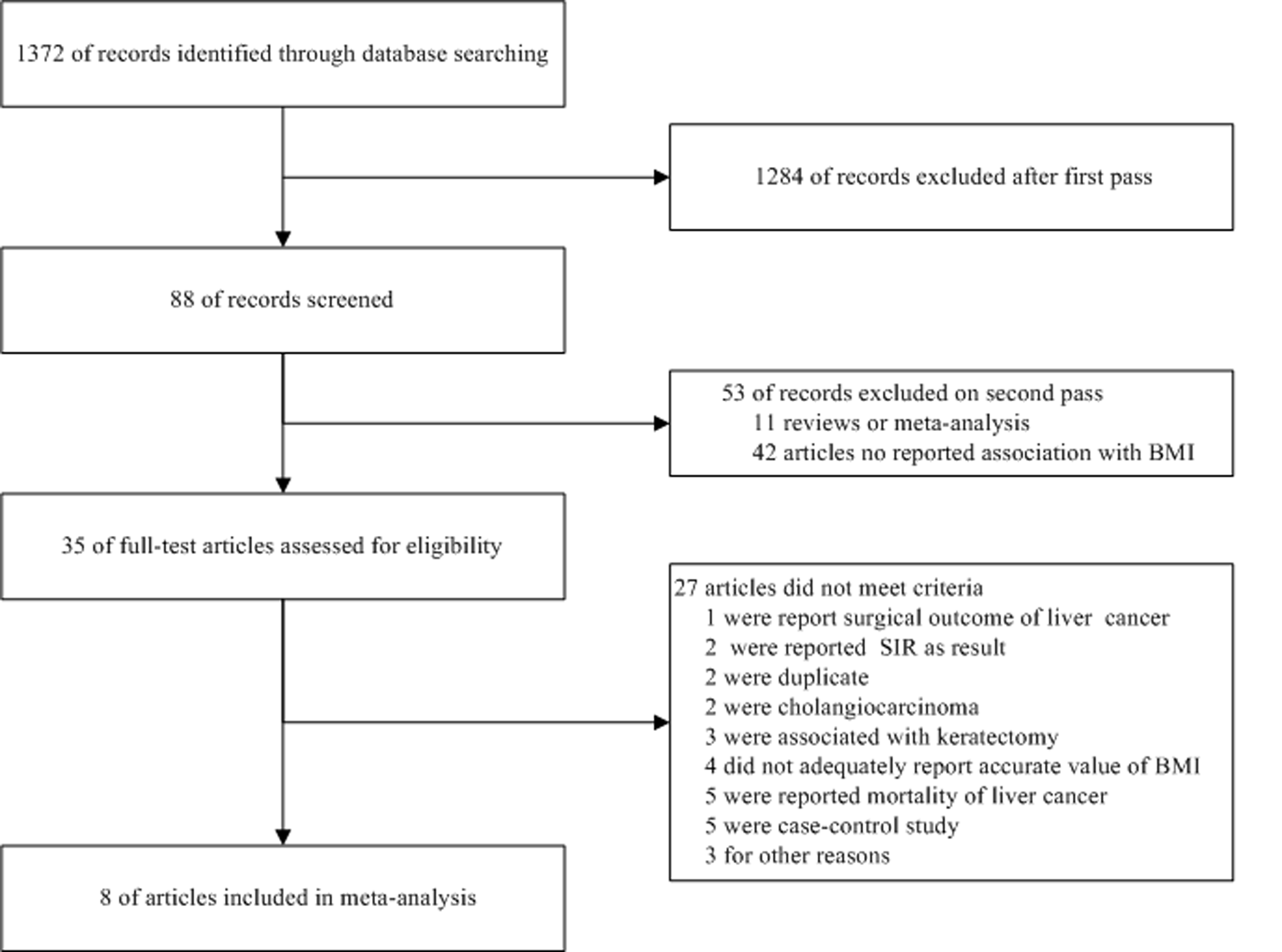

Supplement: Figure S1 — Flow chart of study selection. (TIF) [file pone.0044522.s001.tif]

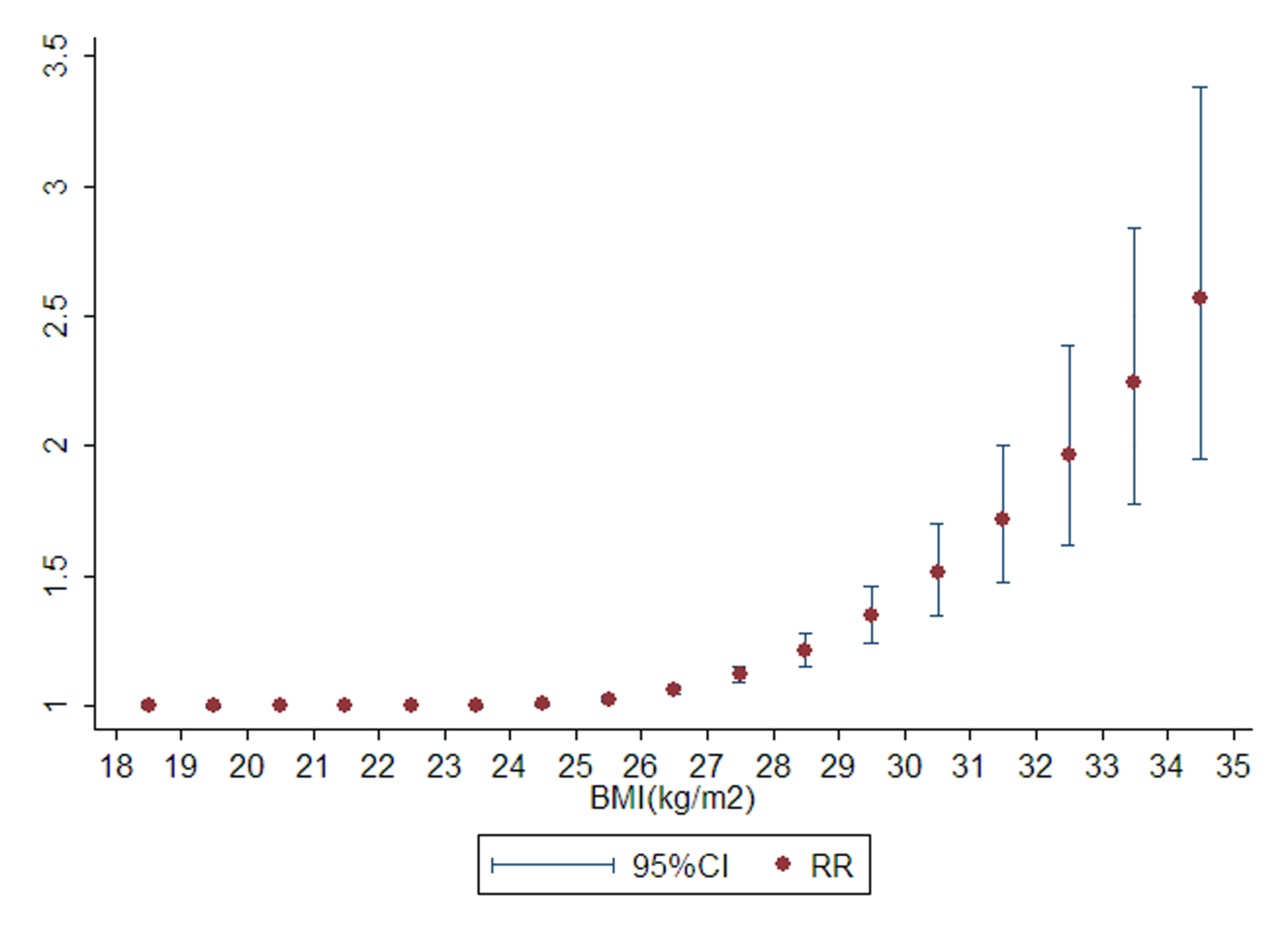

Supplement: Figure S2 — The curve of body mass index and risk of liver cancer for male ( P -heterogeneity = 0.005; P -non-linear<0.001). (TIF) [file pone.0044522.s002.tif]

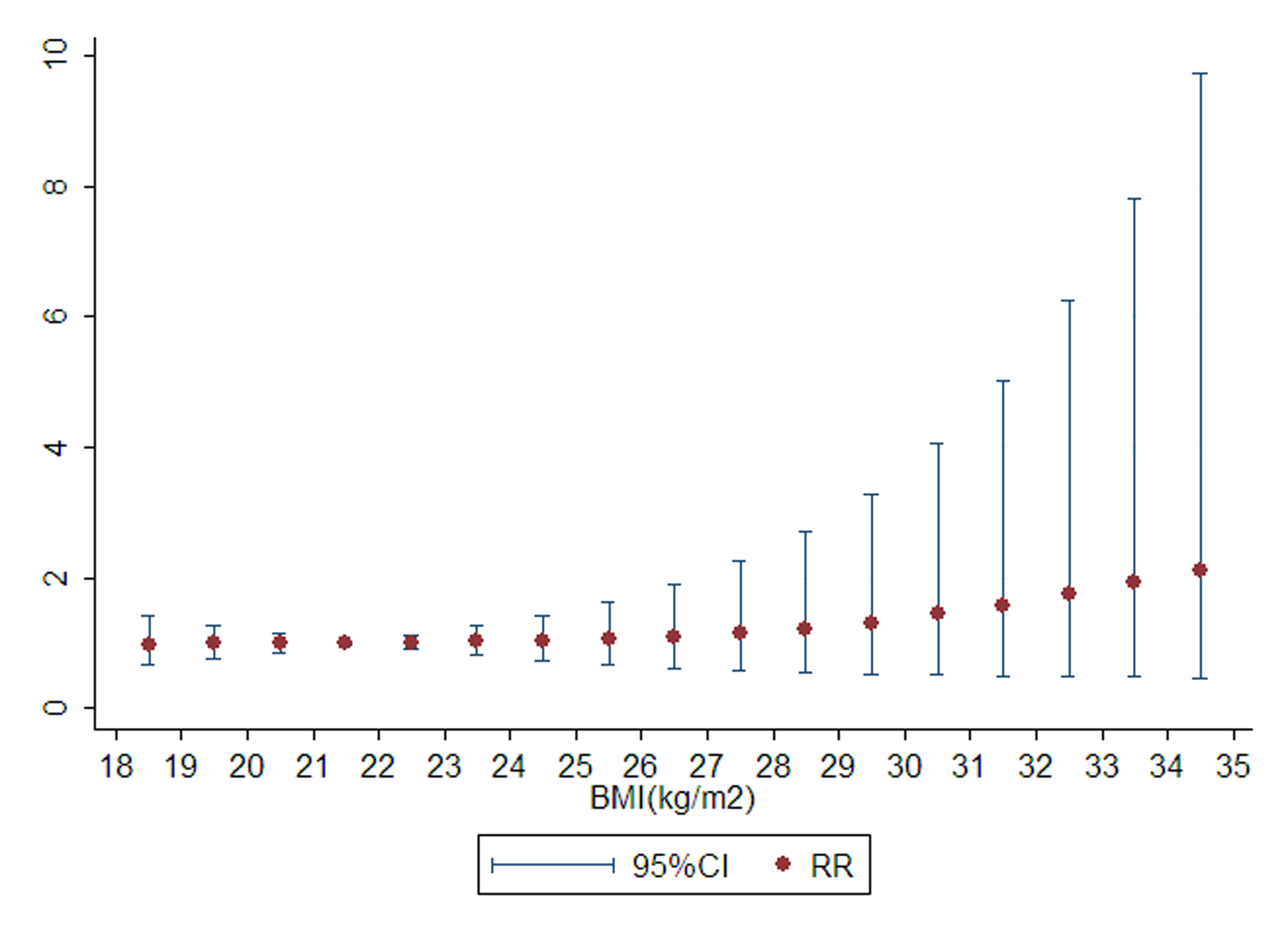

Supplement: Figure S3 — The curve of body mass index and risk of liver cancer for directly measure BMI ( P -heterogeneity = 0.032; P -non-linear<0.001). (TIF) [file pone.0044522.s003.tif]
